# Individualized Repetitive Transcranial Magnetic Stimulation Treatment in Chronic Tinnitus?

**DOI:** 10.3389/fneur.2017.00126

**Published:** 2017-04-06

**Authors:** Peter M. Kreuzer, Timm B. Poeppl, Rainer Rupprecht, Veronika Vielsmeier, Astrid Lehner, Berthold Langguth, Martin Schecklmann

**Affiliations:** ^1^Department of Psychiatry and Psychotherapy, University of Regensburg, Regensburg, Germany; ^2^Interdisciplinary Tinnitus Center of the University of Regensburg, Regensburg, Germany; ^3^Department of Otorhinolaryngology, University of Regensburg, Regensburg, Germany

**Keywords:** chronic tinnitus, repetitive transcranial magnetic stimulation, individualized repetitive transcranial magnetic stimulation, neuromodulation, neurostimulation

## Abstract

**Background:**

Prefrontal and temporo-parietal repetitive transcranial magnetic stimulation (rTMS) in patients suffering from chronic tinnitus have shown significant but only moderate effectiveness with high interindividual variability in treatment response. This open-label pilot study was designed to examine the general feasibility of an individualized fronto-temporal rTMS protocol and to explore what criteria are needed for a more detailed evaluation in randomized clinical studies.

**Methods:**

During the first session of a 2-week rTMS protocol, we applied different rTMS protocols to the left and right temporo-parietal and dorsolateral prefrontal cortex (DLPFC) in 25 tinnitus patients. Short trains of 1, 5, 10, and 20 Hz and continuous theta burst stimulation were applied, and patients were asked for immediate tinnitus reductions after each train. If a patient reported such improvements, rTMS treatment was applied over nine sessions with a combined protocol consisting of the most effective frontal and the most effective temporo-parietal stimulation protocol. Those patients who did not improve after the test session were treated with a standard prefrontal plus temporo-parietal protocol (20 Hz over left DLPFC + 1 Hz over temporo-parietal cortex).

**Results:**

Almost half of the patients (12 of 25) reported immediate tinnitus reductions during the test session. In this group, the mean pre- to post-treatment amelioration in the tinnitus questionnaire was higher (medium to high effect sizes) in contrast to the patients who did not respond to the test session. Treatment outcome remained stable over a follow-up period of 10 weeks.

**Discussion:**

Individualized rTMS was shown to be feasible and effective in chronic tinnitus. The results obtained from this study provide tentative evidence in support of an individualized rTMS treatment approach and might provide a basis for a “tailored” application of rTMS in tinnitus and other neuropsychiatric disorders.

## Introduction

### Chronic Tinnitus

Subjective tinnitus is characterized by the perception of sound in the absence of a corresponding sound source (1). Approximately 1% of the general population report severe tinnitus-related impairment of daily living (2) and seek medical help (3). In contrast to auditory hallucinations that occur in mental disorders and mainly refer to the perception of voices, tinnitus sensations are usually of an unformed acoustic nature such as buzzing, hissing, or ringing (4). Severe tinnitus is frequently associated with depressive symptoms (5), anxiety (6, 7), and insomnia (8), and its socioeconomic relevance is illustrated by the dramatically increased risk for disability pension among tinnitus patients (9). The available evidence-based treatments for tinnitus have only small effect sizes (4, 10, 11), indicating the urgent need for the development and optimization of innovative therapeutic attempts.

### Neurobiological Underpinnings of Chronic Tinnitus

Formerly considered as an otological disorder, tinnitus treatment approaches exclusively targeting the cochlea have led to discouraging results in most cases (12). However, during the last years, advances in neuroimaging and the development of animal models have shifted the perspective toward the neuronal pathologies underlying the different forms of tinnitus (13). There is convincing evidence emerging from functional imaging (14) and neurophysiological studies (15, 16) that tinnitus is not only related to abnormal functioning of the central auditory system (1) but also related to abnormal activity of non-auditory brain regions (17, 18) and abnormal functional connectivity between these regions (19–22). Imaging studies have shown that coactivation of prefrontal areas might especially be related to the affective components of tinnitus (5, 22, 23). It has been further proposed that limbic and paralimbic structures may form a fronto-thalamic gating system for tinnitus perception (24). According to this model, increased neuronal activity arises in the auditory pathways as a consequence of deafferentation due to hearing loss. If altered activity in auditory networks is connected to activation of motivational and emotional brain networks, the inhibitory influence from this fronto-thalamic gating system is downregulated, thus allowing the tinnitus signal to propagate to the auditory cortices, which finally leads to conscious perception. This is in line with electrophysiological studies that demonstrated the relevance of dysfunctional top–down inhibitory mechanisms originating in the prefrontal lobe for tinnitus generation (22, 25, 26). Therefore, two potential targets for brain stimulation can be identified: first, the frontal cortex with the aim to enhance the activity of the fronto-striato-thalamic gating mechanism, and second, the temporal cortex with the aim to reduce cortical activity in the auditory cortex. In line with this notion, tinnitus reduction has been demonstrated after repetitive transcranial magnetic stimulation (rTMS), tDCS, and direct cortical stimulation of both frontal and temporal cortices (27, 28).

### rTMS in Chronic Tinnitus

On the basis of initial findings of abnormal functioning of central auditory structures (1), rTMS of the temporal and temporo-parietal cortex has been proposed as a potential treatment for chronic tinnitus (29). Several clinical studies showed a sham-controlled reduction of tinnitus severity after repeated 1 Hz rTMS applied to the left temporal cortex (30–34), but some studies were also negative (35, 36). In a meta-analysis, treatment effects were shown to be significant, but effect sizes were moderate at best and interindividual variability in treatment response is high (37).

### The *Status Quo*

As mentioned above, rTMS treatment results in chronic tinnitus are currently burdened by only moderate improvement (38) and high interindividual variability indicating the need for optimization strategies. No demographic or clinical criteria could be identified as a predictor for rTMS treatment response in large samples of patients with chronic tinnitus (39). Several studies reported transient tinnitus suppression after a single session of rTMS (40). When the effects of different rTMS protocols over the temporal cortex were compared, it has been shown that the protocol with the best tinnitus suppressing effect differed from patient to patient (41). However, the individually best protocols induced similar changes of oscillatory brain activity (42). Although single rTMS sessions reduced tinnitus only transiently for seconds to minutes, repeated daily rTMS stimulation resulted in longer lasting tinnitus reduction up to several months (30, 43, 44) in some patients. Most studies investigating repeated sessions of rTMS used low-frequency rTMS with 1 Hz, even though two studies comparing the effects of 1 Hz rTMS with those of 10 and 25 Hz revealed similar treatment effects for high-frequency rTMS (45, 46).

In light of these data, the question arises whether a test session, in which the immediate effects of different TMS protocols are evaluated, may be a feasible and useful approach for selecting the rTMS protocol for repeated rTMS sessions. To our knowledge, this question has not yet been addressed by any study, even if the sham-controlled response to single rTMS sessions has been used as a selection criterion for surgical implantation with epidural electrodes (47).

Both auditory and non-auditory brain regions are involved in tinnitus (48, 49), and combined frontal and temporal stimulation protocols exerted best effects in previous studies (22, 50, 51). Thus, the aim of the present study was to investigate the feasibility of an rTMS treatment approach that is (i) individualized based on the effects of single test sessions and that (ii) comprises both prefrontal and temporo-parietal cortices.

## Materials and Methods

### Study Design and Conduct

The study was conducted following a two-armed pilot study design. As a first step, an initial rTMS testing session was conducted in which 1, 5, 10, and 20 Hz; continuous theta burst rTMS; and sham were applied to (i) the left dorsolateral prefrontal cortex (DLPFC), (ii) the right DLPFC, (iii) the left temporo-parietal junction area, and (iv) the right temporo-parietal junction area. The number of applied stimuli during the rTMS testing session on day 1 was 200 stimuli for each stimulation frequency and location (exception: 50 stimuli at 1 Hz frequency), resulting in a total of up to 4,200 stimuli. After the application of each protocol, patients were asked whether they had experienced a change in their tinnitus and were asked to rate the changes on a percentage level regarding to loudness. Patients wore insert earplugs during the testing session and were instructed to report immediate tinnitus changes in form of percentage values. The most effective protocol of each of the four stimulation locations was (i) repeated to assure retest validity and (ii) controlled by sham stimulation (applying the same paradigm with a tilted coil angle of 90° with one wing of the figure-of-eight coil on the head and magnetic field main direction, thus paralleling the scalp surface). Thus, sham conditions were different for each stimulation site and patient during the testing phase. For patients reporting the same tinnitus reduction for different stimulation protocols for the left and right frontal or for the left and right temporo-parietal stimulation sites, equally effective protocols were repeated and contrasted directly. Patients reporting immediate tinnitus modulation during the testing session on day 1 (Monday) were consecutively treated by application of 9 further daily (only working days) combined stimulation sessions consisting of the most effective prefrontal stimulation protocol (2,000 stimuli) at the left or right hemisphere followed by the most effective temporo-parietal stimulation protocol (left or right, 2,000 stimuli/session). Patients who did not experience immediate tinnitus modulation during the testing session on day 1 were treated by a “standard” combined treatment consisting of 2,000 stimuli of 20 Hz rTMS delivered to the left DLPFC followed by 2,000 stimuli of 1 Hz rTMS of the left temporo-parietal junction area (*n* = 3) or a “triple” paradigm consisting of 2,000 stimuli of 20 Hz rTMS of the left DLPFC followed by rTMS (1,000 stimuli left, 1,000 stimuli right) of the bilateral temporo-parietal junction area (*n* = 9) (52). Due to ethical reasons, we switched treatment for the standard group as it turned out during the course of the present study that the triple stimulation is more effective than the former standard treatment (53). The treatment sessions following the testing session consisted of a total of 4,000 stimuli/day for both the individualized and the standard group and were conducted on nine subsequent working days. Follow-up-visits were conducted 2 and 10 weeks after the end of the stimulation period (=week 4 and week 12 visit, respectively).

Stimulation was performed with a 70-mm figure-of-8 (=butterfly) coil (Cool-B65, Magventure A/S, Denmark) in 40 trains with 50 stimuli and an intertrain interval of 25 s (exception: 1 Hz stimulation was administered in one train). The coil was powered by a MagPro ×100 stimulator (Magventure A/S, Denmark). Stimulation was performed at 110% resting motor threshold (RMT) but not higher than 60% maximum stimulator output. RMT was defined as the lowest intensity sufficient to produce left abductor digiti minimi muscle activation (magnetic evoked potentials >50 μV) with a single pulse delivered to the motor cortex in at least 5 of 10 trials and was determined before the testing session on day 1. For treatment of the left DLPFC, the conventional butterfly coil was positioned 6 cm anterior of the left motor hotspot in a sagittal direction with the handle pointing backward in a 45° angle toward the midline (28). Temporo-parietal cortex localization was conducted following a protocol suggested by Khedr et al. (46) positioning the coil between the temporal (T3/T4-electrode location) and parietal (P3/P4-location) (54) according the 10–20 EEG-positioning system (55). Coil positioning was tangential to the scalp with the handle pointing upward.

All participants gave written informed consent after a comprehensive explanation of the study procedures that data were gathered and analyzed for the Tinnitus Research Initiative database (56), which was approved by the Ethics Committee of the University Hospital of Regensburg (Germany, reference number 08/046). All study procedures were carried out in accordance with the approved guidelines.

### Patient Enrollment

Inclusion criterion was subjective tinnitus with duration of more than 6 months. Exclusion criteria comprised objective tinnitus (with a treatable cause), ongoing other tinnitus treatments during or 3 months before study enrollment, the presence of clinically relevant psychiatric comorbidities or unstable medical conditions, history or evidence of significant brain malformation or neoplasm, history of head injuries, cerebral vascular events, the presence of irremovable metal objects in and around the body, pregnancy, alcohol abuse or intake of illicit substances, and history of prior TMS treatment. Patients were recruited for participation in the study after presentation in the outpatient clinic of the Interdisciplinary Tinnitus Centre at the University of Regensburg, Regensburg, Germany. The study was conducted between November 2011 and June 2012.

### Outcome Measures and Data Presentation

The outcome parameters were change in tinnitus distress and loudness measured with the tinnitus questionnaire (TQ; range 0–84) (57) and a numeric rating scale (range 0–10), respectively. We present change from baseline to the week 2 visit to measure immediate treatment effects and also from pretreatment to posttreatment defined as a mean value of the screening and baseline visits (pretreatment) and of the week 2, 4, and 12 visits (posttreatment). In addition, we demonstrate the number of treatment responders defined as a minimal reduction of 5 points in the TQ (58). Further objectives were the assessment of adverse events and safety information at all visits. Data were assessed according to international standards (59) and registered in a tinnitus database following ICH-GCP regulations (60, 61). We contrasted the group of patients who reported changes in tinnitus after a single session with the group of patients with no response.

All data are displayed as mean ± SD if not otherwise labeled. In case of missing data, the last observation was carried forward, and participants who did not complete rTMS treatment (dropouts) were excluded from analysis (*n* = 1). Due to the pilot character of the study, we focus on the feasibility and identification of modifications for future studies (62, 63). For this purpose, we report raw data and basic statistical tests for rough estimation of the efficacy of the tested treatments. All statistical tests were conducted two tailed, unadjusted for multiple comparisons, and a value of *p* < 0.05 was assumed as statistically significant. For group comparisons, we calculated Student’s *t*-tests for independent measures. Effect sizes are reported according to G*Power 3.1.2 (64). Statistical data analysis was performed using IBM SPSS Statistics for Windows, version 22.0 (released 2013; IBM Corp., Armonk, NY, USA).

## Results

### Feasibility

Twenty-five patients were recruited for participation in the study. One participant aborted the treatment after the first day due to headache after finishing the testing session on day 1 (without reporting immediate effects). Exactly 50% of the 24 remaining patients (*n* = 12) reported immediate modulation of their tinnitus percept during day 1 testing procedures and were therefore allocated to the individualized treatment arm receiving combined rTMS of prefrontal and temporo-parietal cortical areas for another 9 consecutive working days. Nine of the 12 patients who perceived a tinnitus change after TMS were able to indicate the change in a percentage value (ranging between 3 and 70%). The other three patients provided only vague estimations like “better” (see Table 1). Two patients experiencing the most pronounced improvement after active rTMS reported similar improvement after sham TMS during the testing session. No other patient reported tinnitus improvement after sham TMS. Also, these patients were assigned to the individualized arm (subject number 1 and 7 in Table 1). Three patients in each of the study groups were under a psychotropic medication (individualized group: 1× citalopram 40 mg/day, 1× venlafaxine 225 mg/day, 1× opipramol 100 mg/day; control group: 1× citalopram 40 mg/day + trimipramine 40 mg/day, 1× agomelatine 25 mg/day, 1× opipramol 100 mg/day).

**Table 1:** **Individual data for treatment with individualized repetitive transcranial magnetic stimulation (rTMS) and average group data for individualized and standard rTMS (each subject was stimulated with 4,000 pulses per day)**.

	Subject order	Kind of prefrontal stimulation	Single-session tinnitus reduction^a^	Kind of temporo-parietal stimulation	Single-session tinnitus reduction^a^	Resting motor threshold (RMT); stimulation intensity (stimulator output)^b^	Change in tinnitus questionnaire (TQ) total score from baseline to week 2 visit^d^	Change in TQ total score from pretreatment to posttreatment^c,d^
Individualized treatment (single subject data)	1	Right continuous theta burst stimulation (cTBS)	70%	Right 5 Hz	50%	42%; 46%	24 (−)	20.33 (−)
	3	Right 10 Hz	“Better”	Left cTBS	“Better”	34%; 37%	7 (−)	2.50 (+)
	5	Left cTBS	40%	Left 10 Hz	40%	43%; 47%	0 (+)	1.17 (+)
	7	Left 20 Hz	50%	Left 5 Hz	40%	46%; 51%	11 (−)	15.17 (−)
	9	Left 5 Hz	20%	Right cTBS	10%	37%; 41%	11 (−)	3.17 (+)
	10	Left 20 Hz	2%	Right 10 Hz	2%	33%; 36%	−3 (+)	−8.33 (+)
	13	Left 20 Hz	“Not sure”	Left 20 Hz	“Good”	50%; 55%	9 (−)	9.33 (−)
	16	Right 20 Hz	30%	Left 5 Hz	100%	57%; 60%	−9 (+)	20.83 (−)
	17	Left 5 Hz	“Shortly better”	Right cTBS	“Shortly off”	30%; 33%	20 (−)	5.67 (−)
	18	Left cTBS	10%	Left cTBS	10%	38%; 42%	3 (+)	9.83 (−)
	23	Left 20 Hz	10%	Right 20 Hz	10%	52%; 57%	10 (−)	8.33 (−)
	24	Left 20 Hz	3%	Left 10 Hz	4%	42%; 46%	3 (+)	5.33 (−)

Individualized treatment (mean ± SD)	n.a.	9/12 left; 5 Hz (*n* = 2), 10 Hz (*n* = 1), 20 Hz (*n* = 6), cTBS (*n* = 3)	n.a.	7/12 left; 5 Hz (*n* = 3), 10 Hz (*n* = 3), 20 Hz (*n* = 2), cTBS (*n* = 4)	n.a.	42 ± 8; 46 ± 9	7.17 ± 9.24 (58% responder)	8.57 ± 8.92 (67% responder)

Standard protocol (mean ± SD)	n.a.	Left 20 Hz	n.a.	Left and/or right 1 Hz	n.a.	43 ± 17; 43 ± 9	3.42 ± 7.04 (42% responder)	1.46 ± 8.48 (42% responder)

*^a^Numbers indicate the percent of tinnitus reduction after a single-session stimulation; verbal statements are indicated by quotation marks*.

*^b^Stimulation intensity was 110% of RMT; for safety reasons, 60% was the upper limit for stimulation intensity; RMT and stimulation intensity were comparable in the standard protocol group as two subjects had thresholds highly above 60%*.

*^c^Visits after treatment (week 2 + week 4 + final visit) minus visits before treatment (screening + baseline)*.

*^d^Responder information in brackets (+ = yes, − = no)*.

**Figure 1:**
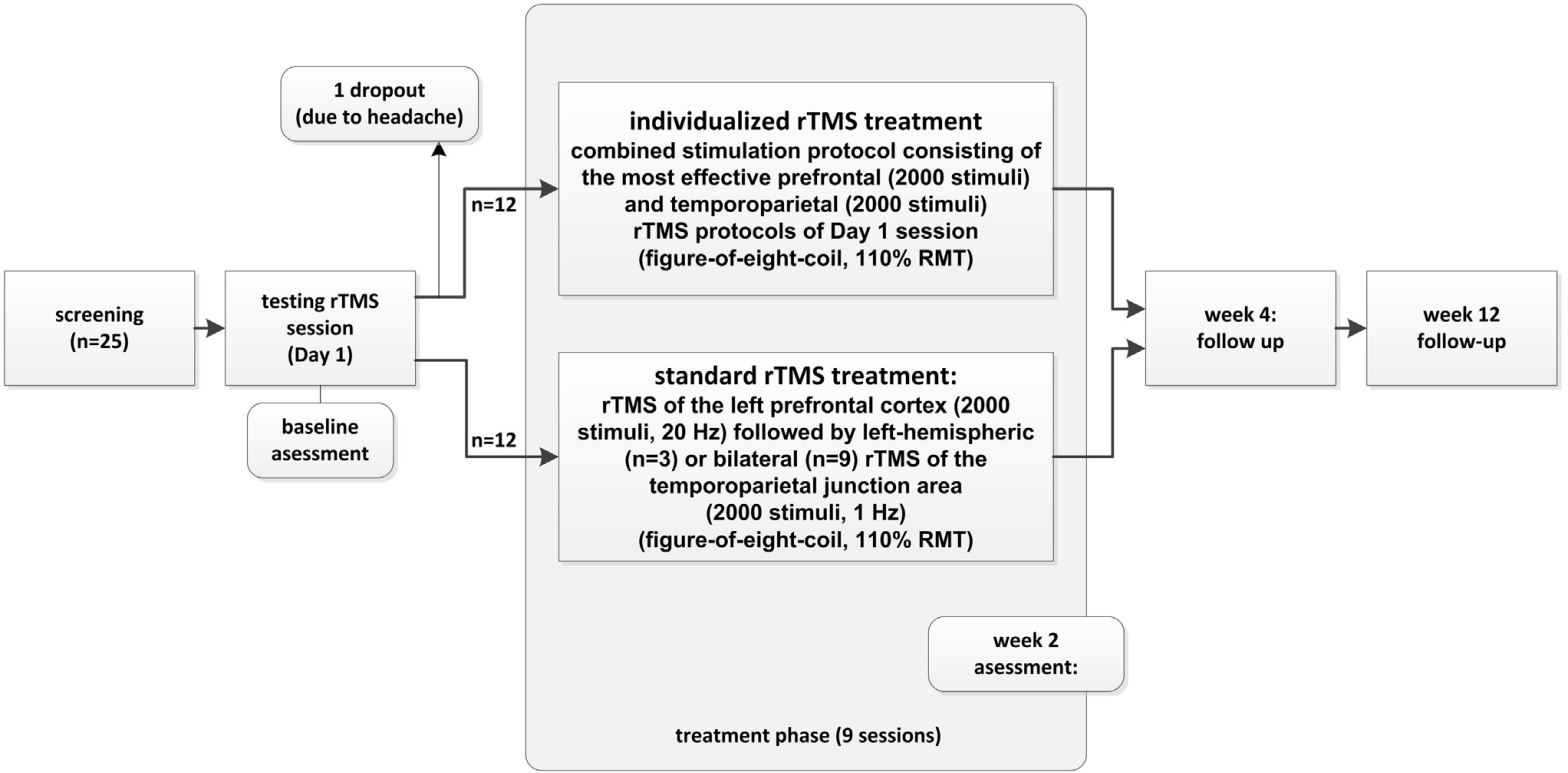
**Study structure and patient flow (for detailed information, see Materials and Methods)**.

Concerning the other 12 patients without immediate tinnitus changes during the testing rTMS session, 3 were treated with the “standard double protocol” consisting of 2,000 stimuli to the left DLPFC at 20 Hz frequency followed by 2,000 stimuli to the left temporo-parietal cortex at 1 Hz and 9 were treated with the “standard triple protocol” consisting of high-frequency rTMS of the left prefrontal cortex (2,000 stimuli, 20 Hz) followed by low-frequency rTMS of the bilateral temporo-parietal junction areas (total of 2,000 stimuli, 1 Hz). For graphical illustration of the study conduct, see Figure 1.

The comparison of participants of both treatment groups revealed no differences in the clinical and demographic baseline characteristics. Detailed information is provided in Table 2. In three patients (all from the individualized group), screening and baseline visits were performed at the same visit resulting in only one pretreatment assessment. One patient did not provide data for visit week 4 (standard treatment). These missing values were added by LOCF using data from the screening visit. For the other patients, interval between screening and baseline was between 13 and 349 days (or screening and baseline was the same visit) with a mean of 81.4 and a SD of 86.03 (median = 53). In the individualized treatment group, we had 10 right-handed patients and 2 ambidextrous patients; in the standard group, we had 8 right-handed patients, 2 left-handed patients, and 2 ambidextrous patients. A statistical comparison with the Fisher’s exact test shows no difference between groups with respect to handedness (*p* = 0.329). In the individualized group, three patients chose right frontal and nine left frontal stimulation as a best stimulation site (χ^2^ = 2.222; df = 2; *p* = 0.045) and five right temporal and seven left temporal stimulation as a best stimulation site (*p* = 0.470), indicating an association of right-handedness with left frontal stimulation. However, this association of left frontal stimulation with right-handedness is from correlational nature, but highlights the need for accounting handedness in rTMS trials.

**Table 2:** **Group comparisons of the two treatment arms**.

	Individualized rTMS (*n* = 12)	Standard rTMS (*n* = 12)	Statistics: individualized vs. standard rTMS
Gender (female/male)	2/10	2/10	n.a.
Age (years)	57.1 ± 7.4	50.6 ± 12.1	*t*(22) = 1.582; *p* = 0.128
Mean hearing level (dB HL)	25.7 ± 15.9	22.9 ± 13.4	*t*(22) = 0.476; *p* = 0.639
Tinnitus duration (months)	108.2 ± 98.9	154.3 ± 106.8	*t*(21) = 1.072; *p* = 0.296
Tinnitus laterality (right/left/both)	0/2/10	0/2/10	n.a.
TQ (baseline minus week 2)	7.2 ± 9.2	3.4 ± 7.0	*t*(22) = 1.118; *p* = 0.276; *d* = 0.465
TQ (screening/baseline minus week 2/4/12)	8.6 ± 8.9	1.5 ± 8.5	*t*(22) = 2.002; *p* = 0.058; *d* = 0.816
NRS loudness (baseline minus week 2)	0.8 ± 1.9	1.4 ± 2.5	*t*(22) = 0.649; *p* = 0.289; *d* = 0.270
NRS loudness (screening/baseline minus week 2/4/12)	0.9 ± 1.9	1.0 ± 1.5	*t*(22) = 0.181; *p* = 0.858; *d* = 0.058

*TQ, tinnitus questionnaire (scale: 0–84); NRS, numeric rating scale (scale: 0–10); rTMS, repetitive transcranial magnetic stimulation*.

### Safety

No severe adverse events occurred during the course of the study. One participant withdrew his consent to participate due to the experience of headache during the testing session on day 1. This patient did not report changes in tinnitus loudness after single rTMS sessions and was determined as dropout for the standard treatment arm (see Figure 1). A deterioration of tinnitus was reported by two patients in the testing session, but did not lead to an interruption of the treatment. These patients were treated with the standard protocol. Further adverse events included headache in two patients (one on 1 day of treatment and one in 2 days of treatment) of the standard treatment and in one patient in the individualized group on 1 treatment day. One patient of the standard treatment missed two treatment days due to a common cold.

### Results of the Test Session

For the majority of patients, the testing session revealed best results for left-sided stimulation for both frontal and temporal stimulation resulting in predominant left-hemispheric treatment location in the individualized treatment group (Table 1). With respect to tinnitus localization, two patients of both treatment groups had purely left-sided tinnitus, and the other patients had tinnitus either in both ears or within the head. No patient had reported purely right-sided tinnitus (Table 2). In the majority of the patients, high-frequency stimulation protocols or TBS revealed best results in the testing session (Table 1).

### Efficacy

**Figure 2:**
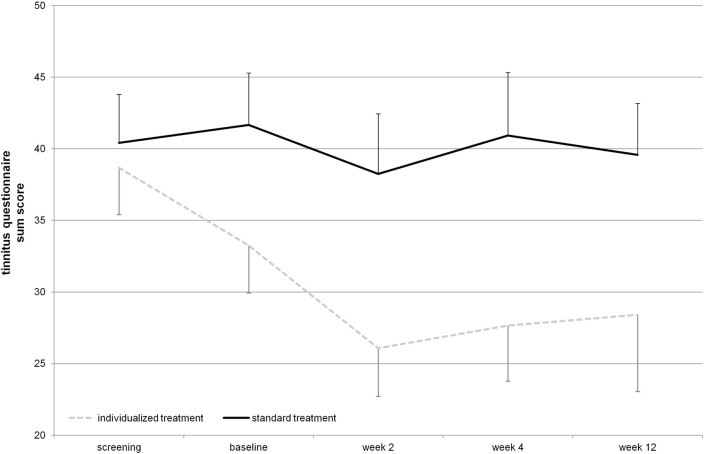
**Tinnitus questionnaire total score over the time course of the study (displayed mean ± SEM)**. Repetitive transcranial magnetic stimulation treatment took place from baseline until week 2 visit. Week 4 and week 12 assessments represent follow-up.

The mean reduction of tinnitus severity measured by the TQ (57) was numerically higher for the individualized treatment group in contrast to the standard group (Table 2). Number of responders was also higher in the individualized group (Table 1). Graphical information on the TQ score at the different assessment time points is depicted in Figure 2. Decrease in tinnitus loudness was in the same range for the individualized and the standard group (Table 2). On a statistical level, individualized treatment (vs. standard treatment) showed numerically superior efficacy with medium or high effect size on a non-significant level contrasting week 2 and baseline or postvisits and previsits, respectively (screening and baseline vs. week 2, 4, and 12) as elicited by the TQ. Changes in tinnitus loudness were not significantly different between groups with small or negligible effects sizes.

## Discussion

From this pilot study examining the feasibility of an individualized treatment approach in patients suffering from chronic tinnitus, three main conclusions can be drawn:

(i) The application of a testing rTMS session with different frequencies and stimulation sites has proven to be feasible with almost 50% of the patients reporting immediate effects in our study sample.
(ii) An individualized treatment approach following this testing session was well tolerated by the majority of patients. Adverse events and dropout rates were comparable among both study arms and matched prior observations (28).
(iii) The clinical efficacy of an individualized treatment approach exceeded the results of a standard treatment approach on a descriptive level. Effects for TQ were not significant, but showed medium to high effect sizes. Improvement was stable for several weeks after the treatment.

This pilot study addressed the question to what extent clinical effects of a single session may serve as predictors for treatment response of repeated sessions of rTMS. On a descriptive level, the individualized treatment group reported a higher reduction of tinnitus severity than the control group treated with a standard combined rTMS paradigm.

Our finding that response to a single session may predict treatment outcome of prolonged stimulation has similarly been detected in the approach to use a single session of rTMS as a selection criterion for the implantation of epidural electrodes for intractable tinnitus (48, 49). Among patients who responded to a single session of TMS, two-third had also long-term benefit from epidural stimulation (48). Notably, epidural stimulation in chronic tinnitus has been only conducted in few cases of intractable tinnitus, and the results should not carelessly be transferred to our sample and settings. However, this ratio corresponds nicely to our finding that most patients who responded to the testing sessions also responded to repeated rTMS application.

Our finding that the effect of testing sessions predicts the effect of daily treatment is of high clinical relevance as rTMS treatment results are characterized by high interindividual variability and no demographic or clinical tinnitus baseline characteristics could be identified as predictors of rTMS treatment response yet (65). The high interindividual variability in rTMS treatment response in previous studies has been mainly interpreted as an expression of the variability of tinnitus pathophysiology (40). If this was correct, it would be plausible that different patients might respond to different stimulation paradigms, thus profiting from individualized “trial-and-error” treatment attempts (65). Even, if the individualized treatment showed higher efficacy in contrast to standard treatment, the SD of TQ change in the two groups was similar for both treatment arms. This indicates a similar variability in treatment response and suggests that individualized treatment does not increase efficacy by reducing interindividual variability.

One discussable issue is the identification of the most efficient protocol in the testing session. In each patient, 20 different protocols were tested (5 different frequencies at 4 different sites). For practical reasons, the intervals between the different protocols were rather short. Investigators were instructed to wait until the tinnitus returned to baseline level before applying the next protocol. Thus, the interval between different protocols was typically in the range between 1 and 5 min. A testing session, therefore, took approximately 1 h per individual, in some cases even longer. However, we are aware that it cannot be excluded that the effects of the different protocols were influenced by after-effects of the preceding protocols. Thus, the increased responsiveness to high frequency and TBS stimulation may have been partly due to the accumulation of stimulation effects as the order of the different protocols was not randomized during the testing session. It could also be the case that it took some time for the individuals to acclimate to the testing scenario. However, it is unlikely that the effects were only driven by accumulation of stimuli or habituation to the testing situation as TBS was always applied as the last protocol, but not every patient reported best improvement with TBS. Nevertheless, future studies investigating individualized TMS should take a potential order effect into account and follow a randomized protocol regarding the sequence of stimulation frequencies and stimulated hemispheres.

A certain dissociation of the effects of a single session and repeated sessions was observed. Although patients reported loudness reductions of their tinnitus during the testing session, daily treatment had only an effect on tinnitus distress but not on tinnitus loudness. Possible reasons might be related to differences in the applied scales for tinnitus loudness (percentage change after a single session vs. 11 graded numeric rating for tinnitus loudness). Furthermore, ratings during a single session were conducted with inserted earplugs, whereas clinical evaluation over the course of the treatment was done without hearing protection obscuring possible changes in tinnitus loudness.

We are well aware that the comparison between the individualized treatment group and the standard treatment group does not enable us to disentangle whether the response in the testing session serves as a general predictor for response to repeated rTMS sessions or whether the individualized protocol was relevant for the good outcome in the individualized treatment group. In other words, the test session might have only served for the identification of responders at the starting point of the rTMS treatment period. To further address this issue, future studies should be conducted in which patients, who respond to TMS testing sessions, are randomized into standard treatment vs. individualized treatment.

Notably, the present study was designed as a proof-of-concept pilot study, which should primarily evaluate the feasibility and tolerability of an individualized rTMS treatment regime in chronic tinnitus. Thus, we are well aware about the limiting factor of the lack of a sham-controlled study group. It cannot be excluded that the improved outcome in the individualized group was driven by non-specific effects. Patients who experienced tinnitus improvement in the testing session might have developed higher expectations with respect to the daily treatment, which could have contributed to the better outcome. However, active control conditions have especially been recommended in rTMS studies due to the inherent limitations of sham conditions (36, 66, 67). For future studies using this individualized approach, we would suggest the splitting of the patients reporting changes in tinnitus perception into two arms—one arm treated with the individualized protocol and one arm treated with a standard protocol. An optimal design would also include a third sham arm (68).

In summary, our pilot data confirm the potential of individualized rTMS treatment as a non-invasive, safe, and well-tolerated method of brain stimulation in the treatment of chronic tinnitus. Descriptive analyses indicate a remarkable superior effect of the individualized treatment in contrast to standard treatment even if the standard treatment with two or three stimulation sites was shown to be more effective than single-site stimulations (36, 66, 67, 69). Individualized rTMS in chronic tinnitus might provide a basis for an individualized, “tailored” rTMS-based therapeutic approach also in other neuropsychiatric disorders. Combining a single session with electroencephalography during the first treatment day (70) might help to identify neuronal markers, which might enable reliable predictions regarding treatment response after daily rTMS. This approach could eventually be useful in identifying successful TMS protocols based on EEG markers also in those patients, who were not able to detect perceptual improvements in the test session.

## Author Contributions

PK, BL, RR, and MS: conception of the study, data interpretation, and manuscript edition. AL and MS: statistics and data management. PK, VV, and TP: patient recruitment and conduction of clinical visits.

## Conflict of Interest Statement

The authors declare that the research was conducted in the absence of any commercial or financial relationships that could be construed as a potential conflict of interest.
